# Exploring Saudi Arabia’s care economy in health, education, and social care: a textual analysis using the care diamond framework

**DOI:** 10.3389/fpubh.2025.1688814

**Published:** 2025-10-20

**Authors:** Atheer Khalid AlSaif, Sama'a Hamed AlMubarak, Faisal Mashel Albagmi

**Affiliations:** Department of Public Health, College of Public Health, Imam Abdulrahman Bin Faisal University, Damam, Saudi Arabia

**Keywords:** care economy, care diamond framework, paid and unpaid care, Saudi Arabia’s care services, textual analysis

## Abstract

**Objective:**

The study delineated the care economy in the context of Saudi Arabia by systematically exploring its structure, coordination, and challenges, focusing on healthcare, education, and social care through Razavi’s Care Diamond framework.

**Methods:**

A qualitative design with deductive textual analysis was employed to analyse 40 sources (34 webpages, 6 policy documents) from the state, market, community, and family sectors. Purposive sampling identified materials most likely to provide policy-relevant insights. Braun and Clarke’s six-phase thematic analysis guided coding, with NVivo used to organize and synthesise sectoral roles, overlaps, and gaps.

**Results:**

The study identified five interrelated themes: (1) absence of a formal care economy structure, with the concept absent from policy discourse; (2) state dominance as the primary architect of care across all domains, but with fragmented coordination; (3) market sector participation concentrated in high-cost healthcare, with minimal education and no social care involvement; (4) family’s critical but unrecognized role, particularly unpaid caregiving by women, indirectly addressed through employment subsidies; and (5) community sector contributions filling care gaps but remaining inconsistent and under-supported. The analysis revealed strong state control but weak integration across sectors, resulting in duplication, inefficiencies, and the marginalisation of unpaid and community-based care.

**Conclusion:**

Saudi Arabia’s care economy is characterised by state dominance, sectoral imbalance, and fragmented delivery. Formal recognition of all four care diamond sectors, integration of unpaid care into policy, and cross-sectoral coordination are essential to achieving Vision 2030 goals.

## Introduction

### Background

Changes in demographics, economic diversification, and the ambitious targets outlined in the Saudi Vision 2030 are transforming care services in Saudi Arabia. Saudi Arabia is the largest country in the Gulf region, with a population of approximately 35.3 million people in 2024 and a Gross Domestic Product (GDP) per capita of 65,880 (1). Despite increased budget allocations to the health sector and improvements in general population health, key health outcomes such as high prevalence of chronic diseases (e.g., diabetes), lower life expectancy, and elevated infant and maternal mortality rates remain below those of other Gulf Cooperation Council (GCC) countries (2). Indeed, gaps in care delivery are driven by the lack of standardised protocols and treatment pathways, which are further exacerbated by variations in delivery, access, and investment that prioritise serving the population over treating patients (3). These persistent gaps in outcomes and service delivery have driven the government to launch major reforms in the health sector, most notably the Health Sector Transformation Program (HSTP), which introduced strategic changes such as privatization and enhanced public–private partnerships, and the National Transformation Program (NTP), which expanded the role of private and non-profit sectors in care provision aimed at improving the health and well-being of the population.

The Health Sector Transformation Program (HSTP) was enacted as an economic action plan as part of the Vision 2030. The Program introduced strategic changes at multiple levels of the Saudi healthcare sector, including privatisation and enhancing public-private partnerships. It also focused on preventive care, person-centred models, and digital solutions such as e-health and telemedicine to improve integration, reduce hospital burden, and expand access, particularly through home-based care and coordinated referral systems (4). Aligned with these reforms, specifically, the National Transformation Program (NTP) targets an increased contribution of the private and non-profit sectors to care provision, from the current 1.8 to 14% by 2030. This is consistent with expanding the private sector’s contribution from 40 to 65% of the national GDP (4). Currently, private healthcare organisations have broadened their involvement in education and professional training through the Capability Development Program, an initiative under Vision 2030 that links the health and education sectors. This focus on building human capital is critical to ensuring that a skilled workforce supports healthcare reforms. The program aims to align academic outcomes with the needs of the healthcare market and build a competent national workforce for delivering quality care (5).

Vision 2030 also prioritises social empowerment by increasing the female participation rate in the workforce to 30%, with a direct implication for caregiving roles and demand for childcare services (6). Similarly, expanding private health insurance coverage is a key strategy, with the government projecting an increase in beneficiaries from 9.8 to 21.7 million through the Council of Health Insurance, which aligns with Vision 2030’s goal of developing private medical insurance to increase access to medical services (4). Collectively, these reforms highlight the government’s commitment to strengthening multi-sectoral coordination as part of Saudi Arabia’s broader transformative agenda.

### Care economy in Saudi Arabia

The care economy refers to the sector of economic activities, both paid and unpaid, that involve the provision of care services, such as childcare, eldercare, and healthcare, which are crucial for individual and societal well-being. It includes direct, relational care activities (e.g., feeding an infant, nursing an ill family member) as well as indirect support such as cooking, cleaning, and organising care. The care economy has direct financial implications in addition to its social role: unpaid caregivers, who are mostly women, face opportunity costs in the form of decreased income and career advancement. Paid care work is often undervalued despite being a significant source of employment. At the macroeconomic level, spending on care services boosts long-term social productivity, encourages labour market participation, and increases GDP growth (7). Globally, the International Labour Organisation (ILO) highlights the gendered nature of care labour, where women bear the greatest burden of unpaid responsibility, which limits their participation in the labour market. In 2023, 748 million individuals aged 15 years or older did not participate in the global labour market due to care responsibilities. Women constituted the vast majority, at 708 million, compared to men, at 40 million (8).

In the Saudi Arabian context, caregiving is family-centric, with women predominantly shouldering the responsibility for unpaid domestic and care work. Extended families play a pivotal role in providing elder care and childcare. According to the United Nations Women Arab States, paid care employment in health, social care, and education accounts for 12 to 18% of the total employment (9). These cultural norms are now shifting as sociocultural dynamics change. Female participation in the workforce has increased significantly, and family structures and caregiving roles, particularly those involving children, are transforming. Vision 2030 has empowered women, increasing their involvement from 17 to 35% between 2017 and 2024, which has led to a higher demand for childcare and older population support (10). However, persistent gender role expectations and uneven access to affordable care services still limit women’s full economic participation. Although recent interventions such as the Qurrah Subsidy Program, which supports working mothers by subsidising childcare costs, and the Wusool transportation program, which indirectly contributes to the care economy by enabling women to remain in the labour market while balancing family caregiving responsibilities, seek to address sociocultural barriers, challenges of adjustability and equitable reach remain. However, unlike Qurrah (11, 12). The Ministry of Human Resources and Social Development (MHRSD) provides comprehensive care for the older population, including health, social, and psychological services, through social care homes distributed across the Kingdom. Additionally, it provides financial and in-kind support to older population individuals in need. These initiatives highlight the state’s role in the care economy as both a direct provider of institutional care and a financier of elder support, complementing but not replacing the unpaid care traditionally delivered by families (13).

Alongside these social shifts, Saudi government efforts to fund and expand care delivery have contributed to the proliferation of care facilities encompassing hospitals, rehabilitation centres, orphanages, and daycare institutions, suggesting progress in care delivery. Several ministries, including Health, Education, and Human Resources and Social Development, operate these care facilities, while private providers and non-profit organisations complement government efforts by filling the remaining service gaps (14, 15).

Despite these developments, understanding how such a diverse mix of providers interacts and contributes to the overall care system requires situating Saudi Arabia’s experience within broader theoretical and empirical considerations of the care economy. Theoretical discussion of the care economy has largely emphasised its role in supporting labour markets and socioeconomic well-being (16, 17). Yet, the majority of empirical studies focus on Western contexts, paying little attention to the GCC countries, including Saudi Arabia (18). Saudi Arabia lacks empirical research, with data limited to policy and program evaluations, such as Vision 2030 and ministerial reports; however, it lacks scholarly analysis of how care provision is structured, coordinated, and accessed in practice (19). Existing literature emphasises macro-level trends, such as growing female labour force participation (20); however, it neglects micro-level policy interactions with culture and care labour (15).

The Saudi context offers unique dynamics that are not well covered in the literature, such as an extensive reliance on unpaid family caregiving, restrictive gender roles, and an unequal distribution of the private sector between urban and rural areas. These distinct features reinforce the necessity of addressing current empirical research gaps and emphasise the need for context-specific, data-driven research to align Saudi Arabia’s care economy with reforms that promote equitable access, efficient coordination, and improved care outcomes.

### The care diamond framework

The care diamond framework, developed by Razavi, examines care services through four key sectors: state, market, community, and family (17). In Razavi’s work, the term “architect of care” refers to the structural design, or “care diamond,” that organises the provision and financing of care among these four key sectors. This architecture determines who is responsible for delivering care, especially for those with intensive needs such as children, the older population, and people with disabilities. Razavi emphasises that while each sector plays a role, the state holds a qualitatively distinctive position as the key coordinator, regulator, and policy decision-maker. It shapes how care is distributed across the other sectors by determining priorities, setting eligibility rules, and designing and implementing care policies. Thus, the “architect of care” reflects the institutional structure through which care responsibilities are assigned and managed within a society.

The framework’s strength lies in its ability to map interdependencies, such as how state subsidies intersect with private insurance or familial caregiving. The framework has been applied in analysing systemic fragmentation and gender inequities in care provision (21, 22). For instance, in Japan, Abe utilised the care diamond framework in a qualitative, analytical case study to compare childcare and eldercare policies, revealing structural differences and persistent gender inequalities (22). In Serbia, Perišić & Pantelić conducted a theoretical review using the care diamond framework to examine the evolution of care policies. The study highlighted sectoral gaps, particularly the limited contribution of the community sector in childcare, showing that eldercare formed a complete care diamond, while childcare resembled a “care triangle” (21). Similarly, Ochiai used the framework in a comparative interpretive study across six East and Southeast Asian countries, reinterpreting existing empirical data to map care networks and categorise welfare regimes (23). These applications demonstrate the framework’s utility in revealing institutional and sectoral fragmentation, gendered care patterns, and varied care arrangements across global contexts.

The application of the care diamond framework remains limited in the Middle East. However, Duffy et al., applied the care diamond framework in a quantitative comparative study of 47 countries, including Middle East and North Africa (MENA) countries such as Egypt and the State of Palestine, to examine how care responsibilities are shared across the state, market, family, and community sectors and, to analyse the structure and size of the paid care workforce (24). The framework was used to examine how care provision varies across regions and its relationship to economic development, gender equity, and care needs. The study also emphasised that while the care diamond is a valuable conceptual tool, its paid components vary widely depending on national policies, labour market structures, and care demand.

While the care diamond framework has been applied in global contexts, including Japan, Serbia, and MENA countries such as Egypt and the State of Palestine, no studies to date have applied this framework within the context of Saudi Arabia. This highlights a significant gap in the literature, underscoring the need for empirical research that adopts the care diamond to examine care provision and coordination, as well as the distribution of responsibilities across sectors in Saudi Arabia. Given the aim of the current study, the care diamond provides a practical framework to examine care delivery in Saudi Arabia while providing a holistic analysis of roles, overlaps and gaps across all four sectors.

### Research aim

The study delineated the care economy in the context of Saudi Arabia by systematically exploring its structure, coordination, and challenges. Specifically, the study used the care diamond framework to examine the interactions between the state, market, family, and community as the four pillars of the care economy (25). This examination was conducted within the domains of health, education, and social care^1^ in Saudi Arabia, as they are widely recognised in the literature as core domains of the care economy (26). Their prominence in the literature reflects their role as institutional bases through which governments deliver formal care to children, the older population, individuals with chronic conditions, and other vulnerable populations (27). They are also responsible for policy direction and direct or indirect care delivery, and are critical in sustaining population wellbeing and economic productivity. Peng reports that healthcare, education, and social care are among the fastest-growing areas of employment globally, underscoring an increasing demand for care services (27). Shifting demographics and evolving norms in care delivery make the three domains key policy priorities and targets for investment. Hence, focusing on healthcare, education, and social care ensures this study captures the institutional backbone of the care economy and supports policy-relevant insights.

Additionally, the selection of the three care domains is further justified by their fundamental role in shaping and delivering care in Saudi Arabia, where a clear, integrated care system that coordinates healthcare, education, and social care providers under a unified framework is still lacking. The three ministries —Health, Education, and Human Resources and Social Development — are the most directly involved in formal care provision and represent the governmental institutional response to care needs (14). Healthcare plays a vital role in ensuring the physical and mental well-being of individuals, while the education sector trains healthcare professionals and shapes social attitudes toward caregiving. Furthermore, social care services provide essential safety nets for vulnerable populations such as the older population and individuals with disabilities (28). Examining these domains together offers a holistic perspective on how the Saudi government structures, delivers, and regulates care. This approach also enables mapping the existing care landscape, identifying policy gaps and challenges, and uncovering opportunities for future development and investment in the care economy. To understand how these three domains operate within the broader care system, this study applies the care diamond framework, which situates care provision across four key institutional sectors: the state, the market, the family, and the community (25).

### Research objective

To examine the distribution of care responsibilities across the state, market, family, and community sectors in Saudi Arabia by assessing the level of coordination and integration among these key providers, particularly in healthcare, education, and social care, using Razavi’s Care Diamond framework.

### Significance of the study

The study’s findings offer actionable insights to align healthcare, education, and social services with Vision 2030’s goals. The research also identifies operational, financial, and cultural insights to provide a foundation for policy reforms that enhance sector integration, maximise resource allocation, and strengthen social protection systems. These collectively contribute to building an inclusive, resilient care economy aligned with Saudi Arabia’s social and economic ambitions. The study also expands the existing literature on the care economy by examining it in the non-Western context of Saudi Arabia, with a particular focus on its unique cultural and social aspects. Furthermore, it extends the application of the care diamond framework in the context of Saudi Arabia. Such application and analysis enrich the currently scarce literature and allow for future comparative studies.

## Methodology

### Research design

The current research adopted a qualitative design to delineate the care economy in the context of Saudi Arabia, with the care diamond as a guiding framework. Qualitative research aims to understand a social phenomenon in detail by collecting and analysing non-numeric data, which is ideal for exploring meanings, experiences, and interpretations rather than cause-and-effect relationships (29). This approach enabled the researcher to explore in detail how the sectors relate, coordinate, and deliver care services, as well as the socioeconomic impact on beneficiaries. The context-specific nature of qualitative research also enabled a focused exploration of institutional documents, official webpages, policies, and frameworks that define care provision (30), while its flexibility allowed for the incorporation of new information during data collection and analysis (31). It also facilitated the identification of themes and patterns, offering a structured and interpretive understanding of care coordination across sectors in Saudi Arabia.

### Data collection

This study employed a purposive sampling strategy to identify policy documents and official webpages relevant to Saudi Arabia’s care economy. This non-probabilistic approach enabled the selection of sources most likely to yield rich insights into how the state, market, community, and family sectors conceptualise, govern, and operationalise care services. Data collection for textual analysis covered two types of sources: formal documents and relevant webpages from governmental and non-governmental entities.

Key entities included ministries, regulatory bodies, private care providers, and community organisations, specifically, the Ministry of Health (MoH), Ministry of Education (MoE), and Ministry of Human Resources and Social Development (MHRSD). Each ministry’s official website was systematically explored to locate strategic plans, regulations, programs, and policy reports that either directly addressed or indirectly referenced care-related issues. This was complemented by an extensive online search using Google to capture additional materials with keywords such as “care economy,” “unpaid care,” “childcare,” “eldercare,” “social care,” “home care,” “community-based services,” “private sector health investment,” “Vision 2030,” “Saudi Arabia care services,” and “family caregiving support programs.”

The search yielded 40 sources, including 34 webpages and 6 documents. These 40 sources were selected as they represent the most recent, authoritative, and strategic documents and webpages issued by key ministries and recognised organisations. Priority was given to official publications and widely circulated webpages that explicitly or indirectly address care-related issues. This ensured that the dataset captured the most relevant and policy-influential materials rather than outdated or peripheral sources. The dataset comprised 27 state sources, including ministry reports, regulations, strategic plans, and national initiatives (see Supplementary Table 1). The analysis also included seven market sources, comprising private healthcare providers, and Public-Private Partnership (PPP) announcements, as well as six community sources, including Non-Governmental Organisations (NGOs), civil associations, and community-private sector partnership initiatives (see Supplementary Tables 2 and 3, respectively). Of the 34 webpages, 19 were obtained from the state, seven from the market, six from the community and two from the family sector; however, the latter were state-produced documents concerning family-related issues, underscoring that the family’s voice is mediated through state platforms. As shown in Table 1, the state sector webpages covered healthcare, education, and social care; the market focused mainly on healthcare; the community spanned all three domains; and the family sector had limited representation.

**Table 1:** Distribution of webpages (*n* = 34) across care diamond sectors and their focus on healthcare, education, and social care.

Care diamond sector	No. of webpages (34)	Healthcare	Education	Social care
State	19^1*^	5	8	8
Market	7	6	1	None
Community	6	3	3	
Family	2	None	None	2
Total	34			

^1*^The total number of unique state webpages is 19. Two webpages addressed both education and social care; therefore, they were counted in both categories, which makes the sum across columns appear higher than the total.

Among the six documents, the Vision 2030 report and the King Khalid Foundation report addressed all three domains. The Healthcare Sector Transformation Program (HSTP) and the Healthcare Strategy in the Kingdom focused on healthcare. The Manual of Women’s Employment in the Private Sector emphasised social care, while the Life Skills Guide covered both healthcare and social care. Of these six documents, five were state-affiliated, and one was classified under the family sector; none were obtained from the market or community sectors. A complete list of analysed sources, categorised by sector and domain, is provided in Supplementary Tables 1–3.

### Data analysis

Through textual analysis, the study examined the retrieved webpages and documents to understand how the care economy is conceptualised and organised in Saudi Arabia. Textual analysis is a qualitative investigation that systematically interprets texts to discover underlying themes and derive meanings (32, 33). Textual analysis of institutional documents provided a clearer understanding of how the different sectors of the care economy, as defined by the care diamond framework (19), function and coordinate to meet the needs of beneficiaries. Given the established body of literature, the study adopted a deductive approach using the care diamond framework to guide this analysis, which revealed sectoral roles and overlaps. This framework proposes four key sectors: state, market, community, and family, each contributing to holistic care (25).

Braun & Clarke’s six-phase framework guided the thematic analysis. First, a total of 40 webpages and documents were reviewed to familiarise with socioeconomic and regulatory details of care delivery (34). Second, initial codes were generated deductively, which organised data into categories aligned with the care diamond framework (e.g., paid caregivers coded under “market”; public care and social security programs under “state”; community-based initiatives under “community”). Third, codes were clustered into broader themes based on study objectives and the care diamond’s sectoral interdependencies. Fourth, themes were reviewed by cross-referencing codes with original documents to ensure consistency. Fifth, themes were refined with the inclusion of explicit examples from the data. Finally, themes were synthesised into a cohesive narrative, integrating direct quotes and appendices of sector-specific services. NVivo version 15 facilitated the data analysis process (35).

### Trustworthiness

Rival explanations were methodically considered during the research to challenge presumptions and encourage a fair interpretation of the findings. The lead researcher conducted the coding and analysis using NVivo software, with coding decisions and emerging themes regularly reviewed and discussed with the research supervisor to ensure consensus and to consider alternative interpretations. The researcher critically evaluated whether each data excerpt fit the sector initially assigned to it —state, market, family, or community — or whether alternative interpretations were plausible. Before final coding decisions were made, overlapping responsibilities — such as cases where both state and market sectors could influence care provision —were examined in light of the care diamond framework. As a Saudi researcher with a background in public health and women’s health, the researcher had an understanding of the cultural norms and national policy context about caregiving. Although this knowledge informed the interpretation of texts, it also necessitated thoughtful reflection to reduce any potential bias. While this knowledge-informed interpretation, reflexivity was maintained to acknowledge that such familiarity could also introduce bias. To minimise this risk, coding decisions were revisited and systematically checked against both the dataset and the care diamond framework, ensuring that interpretations were grounded in evidence rather than personal assumptions. Throughout the analysis, careful attention was given to aligning interpretations with the care diamond framework and to revisiting coding decisions to ensure they were based in the data rather than personal assumptions. This approach strengthened the study’s credibility, analytical rigour, transparency, and overall trustworthiness.

## Results

Textual analysis revealed five interrelated themes that describe the sector’s roles, the system’s dynamics, and gaps in structures that shape the care economy in Saudi Arabia. (a) absence of a formal care economy structure; (b) state as the primary architect of care; (c) private sector participation in care; (d) the family’s critical but overlooked role in care provision; and (e) the community’s role in filling care gaps is supportive yet inconsistent. The results highlighted a disintegrated care system, characterised by a predominance of the state sector, limited market and community input, and a lack of formal recognition of the family sector.

### Theme 1: absence of a formal care economy structure

The term care economy was absent from almost all the 40 analysed documents and webpages. The King Khalid Foundation report, produced by a non-governmental organisation, was the only document that explicitly recognised the care economy in Saudi Arabia. Within Razavi’s care diamond framework, it fits within the community sector, while also indirectly engaging the state sector by assessing government initiatives and suggesting regulatory changes. The report plays a cross-sectoral role, comprehensively addressing the three core care domains: health, education, and social care. It reviews both local and global care economy indicators and highlights Vision 2030’s role in enhancing health, social, and educational services in the Kingdom. While the textual analysis reflected the broader absence of the care economy as a recognised model in national policy discourse, some aspects of current initiatives align with core principles of the care economy.

The findings revealed that the state sector webpages and documents (n = 27) predominated the sample, especially those produced by the Ministry of Health, Ministry of Education, and Ministry of Human Resources and Social Development. Analysis showed that while several state documents, particularly from the Ministry of Health, indicated partial alignment with care economy principles, such as efforts to coordinate care providers, promote person-centred services, and deliver targeted support to vulnerable populations through home-based healthcare and e-health initiatives. However, none of these explicitly framed such efforts within a care economy perspective. These initiatives, mainly coded under the state sector and covering the healthcare domain, were implemented without conceptualising care as a shared responsibility among the four sectors outlined in Razavi’s care diamond.

Similarly, sources analysed from the market sector (n = 7), mainly webpages from private healthcare providers, were mostly concentrated in the healthcare sector, with minimal coverage of education and no representation in social care, indicating limited cross-sectoral engagement. These sources focused on delivering premium home care and medical services, which were framed as commercial products prioritising convenience, clinical excellence, and personalisation. However, they frequently overlooked the broader social and economic significance of care, such as its role in promoting family well-being, supporting women’s participation in the labour market, and reducing long-term public health burdens. Instead, care was treated only as a health service to be purchased and sold. This indicated a limited conceptualisation of care, restricted to a market transaction model. Based on the sources analysed, the market sector was underrepresented compared to the state sector, indicating limited policy engagement from the private sector in care provision.

The results of the community sector’s sources (n = 6) showed that services were often presented as charitable efforts rather than being components of an integrated care infrastructure. For example, one webpage described community services as providing “a safe and supportive environment for girls to learn,” emphasising localised support without policy-level integration. These webpages were primarily coded under the education and social care domains, yet they presented standalone initiatives with little indication of coordination with state, market, or family care systems. The absence of the care economy from formal governmental policy across state, market, community, and family sector sources reflects not only a lack of formal structure and policy, but more fundamentally, a lack of its conceptual realisation and integration as a strategic resource to support Vision 2030 goals of building a thriving economy and vibrant society. The absence of the care economy as a conceptual and policy framework contributed to fragmented care provision, with services lacking coordination across health, education, and social care, rather than being recognised as interconnected components of a unified system essential to overall well-being. As a result of this absence, unpaid care work, community-based initiatives, and private sector contributions are often overlooked in national planning, strategies and policy documents.

### Theme 2: the state as care architect: from provider to regulator

The state is the primary architect and dominant sector in the provision of care in Saudi Arabia, shaping and delivering services across healthcare, education, and social care. Across the 40 reviewed documents and websites, 27 were state-based, confirming the government’s central role. Among the 27 state sector documents and webpages analysed, seven focused on healthcare, eight on education, and 10 on social care. In addition, two cross-sectoral documents addressed all three care domains: the Vision 2030 report and the King Khalid Foundation report. This distribution indicated that the state’s involvement in care provision spanned across all key domains. Despite this broad coverage, the analysis revealed a lack of clear coordination mechanisms across sectors, resulting in fragmented care provision and service delivery. This was evidenced by the reviewed sources, which indicated overlapping responsibilities, limited integration, and inconsistent program implementation. For example, documents showed that while the state formulated policies, such as the *Vision 2030 Privatisation Program,* to expand private sector involvement in healthcare, private healthcare providers such as Adeed and Fakeeh, which offered integrated home medical services, operated independently of state oversight. Similarly, both the Ministry of Health (MoH) and the market through healthcare private providers were involved in delivering older population care and vaccination services, without joint planning, leading to duplicated efforts between state and market sectors. Community-led initiatives, such as the National Home Health Care Foundation (NHHF), provided care for patients with chronic conditions yet remained disconnected from the formal healthcare system. These examples showed that private and community projects operated in parallel rather than being integrated into the state’s broader reforms. The disconnection between the state, market, and community sectors reflected a greater focus on policy development than operational coordination, resulting in fragmented care provision. Despite the observed coordination gaps, the textual analysis revealed that the state maintained an active and multifaceted role in care provision, demonstrating both regulatory supervision and direct service delivery across healthcare, education, and social care.

In the healthcare, the state is primarily represented by the Ministry of Health (MoH). MoH acts as both a regulatory and decision-making authority, as well as a direct provider of healthcare services, consistent with its dual role in Razavi’s care diamond framework. Several analysed sources demonstrated the MoH’s role as a regulator through its development of national policies, allocation of public funding, and establishment of strategic priorities to enhance care delivery. One example is the integrated care system promoted in MoH sources, which emphasises person-centred care to ensure treatment aligns with patients’ social, cultural, and medical needs. This approach ensures that care delivery remains flexible and responsive to beneficiaries’ expectations without compromising quality. To operationalise this, the ministry introduced referral system improvements and multidisciplinary teams to coordinate care delivery. Simultaneously, the state directly delivers care, particularly to vulnerable groups such as the older population, people with disabilities, and those with chronic conditions. For instance, Several MoH sources highlighted the home-based healthcare program as a key initiative targeting vulnerable populations. One webpage described it as providing “medical and nursing care at patients’ residences to ensure continuity of care and reduce pressure on hospitals” (36). E-health initiatives were also promoted, with one strategy document stating that the goal was to “enable remote consultation, diagnosis, and follow-up without the need for physical visits” (37). Telework was introduced as a mechanism to enhance labour participation, particularly among women and people with disabilities, by “creating remote job opportunities that overcome barriers such as transportation, workplace discomfort, and caregiving responsibilities” (38). Additionally, government-led vaccination reminder services, including mobile-based systems, are used to support families in ensuring that children receive timely immunisations.

In the education, the Ministry of Education (MoE) documents and webpages showed the state’s role in both service delivery and regulation. For example, the establishment of the School Health Affairs Department was described as a move to “create a safe and healthy school environment through regular health education and preventative services,” as stated in the reviewed sources (39). The analysis also highlighted the Ministry’s commitment to gender inclusion through initiatives that empowered women by expanding access to the education workforce and leadership roles, such as the appointment of the first female spokesperson for General Education in the Kingdom. Additionally, women’s education was linked to labour market readiness, with one document emphasising that the Ministry aimed to ‘sponsor women’s education in fields aligned with market demand’, meaning that women were encouraged to pursue disciplines that matched labour market needs rather than those traditionally chosen. In the social care, documents from the Ministry of Human Resources and Social Development (MHRSD) outlined a network of welfare programs targeting various vulnerable groups. For example, one of the MHRSD reports stated that the ministry “provides integrated services for orphans, the older population, and juveniles through specialised care institutions across the Kingdom” (40). These programs were framed as part of the state’s broader objective to support citizens at various stages of their lives, ensuring they enjoy a decent standard of living.

The textual analysis also revealed a strategic shift in the state’s role from direct provider to regulator, especially in alignment with *Vision 2030* goals. Several state documents highlighted this transition. For instance, the *Health Sector Transformation Program* (HSTP) emphasised a sector-specific shift in the Ministry’s role, stating that the Ministry would “empower the private sector to deliver services while focusing on regulation and quality oversight” (4). Likewise, analysis of the *Vision 2030* report and its associated transformation programs outlined a broader national target across multiple sectors, seeking “an increase in the contribution of the private and non-profit sectors from 1.8 to 14%” in service delivery, aiming to “reduce the government’s operational burden and enhance sustainability” (19). This shift from direct provision to regulation reflects a broader policy transition aimed at enhancing efficiency and achieving long-term sustainability. Collectively, the analysis demonstrated that these initiatives illustrate the state’s multifaceted role in the care economy as regulator, funder, and direct provider of care.

### Theme 3: private sector participation in care

The textual analysis of private sector sources (n = 7) revealed that six webpages focused exclusively on healthcare services, while just one referenced education, and none addressed social care, indicating limited private sector engagement beyond health.

Within healthcare, the analysed sources consistently highlighted the private sector’s provision of specialised, high-cost services, which were marketed as ‘tailored to meet all patient needs with around-the-clock access’. Such market-driven care solutions positioned the private sector as an efficient alternative to public healthcare by focusing on convenience and exclusivity. For instance, one webpage described its services as ‘specialised care delivered with convenience and clinical excellence’. This demonstrated how private healthcare services are frequently marketed in a commercial sense. The reviewed sources also showed variation in the types of care provided. For example, Saba Medical provided a broad set of services, including newborn care, home nursing, older population care, and diagnostic support, such as laboratory testing and radiography (41). Fame Medical focused on maternal and child health through home-based pregnancy monitoring, birth assistance, caesarean care, and breastfeeding support (42). Similarly, the Enfield Royal Clinic offered personalised older population care, including home-based companionship and social support (43). Across these examples, services were often presented as premium, patient-centred solutions that emphasised convenience and individualisation. However, the way they were marketed in the reviewed sources also highlighted inequalities in affordability and accessibility, suggesting that private care was positioned as a specialised option rather than an integrated part of broader care provision.

Although private sector services aligned with Vision 2030’s goal to expand private participation in healthcare, the textual analysis showed that their involvement remained limited and concentrated in specialised, high-cost care. These services, such as personalised and premium older population care, were inaccessible primarily to underserved populations and were presented in the reviewed sources as operating independently of state systems. This lack of integration highlighted fragmentation between the state and market sectors, with each functioning in isolation rather than as part of a cohesive care system.

In education, the analysis of market sector sources revealed limited private sector participation, with involvement primarily focused on infrastructure and innovation rather than direct service provision. One document described partnerships between the Ministry of Education and private actors aimed at “sharing resources and expertise to improve learning environments” and “promoting investment in research and educational infrastructure” as part of a long-term strategy to enhance the quality of the system (44). Additionally, the Ministry introduced regulations for international and private schools to improve outcomes and streamline procedures, thereby expanding private educational provision and offering families more quality schooling options to support child development and well-being. While the education sector demonstrated emerging efforts to build public-private partnerships, the reviewed material confirmed that market sector involvement remained overwhelmingly concentrated in the healthcare sector.

Social care was absent from the analysed market sector webpages. None of the reviewed sources indicated market sector involvement in older population support, disability services, family caregiving, or community well-being initiatives. This absence suggested that the market sector was not engaged in supporting vulnerable groups beyond profit-based medical services. Overall, the findings from the analysed sources indicated that the private sector’s contribution to care remained limited, primarily commercial, and health-oriented. The examined sources demonstrated that, despite Vision 2030’s policy emphasis on expanding private participation, market-based care services were not generally available and frequently lacked integration with public systems. The Vision’s stated goal to increase and control private sector contributions to provide better equity and sustainability in care services may be explained by this limited and fragmented involvement.

### Theme 4: the family’s critical but overlooked role in care provision

The textual analysis found that the family’s role in care provision, although central in practice and a core pillar in the care diamond framework, was absent from the reviewed official governmental documents. None of the sources, including those issued by the Family Affairs Council (FAC) and the MHRSD, explicitly acknowledged family caregiving. No documents identified the family as a formal care provider or outlined policies to support unpaid care within the household. This absence revealed a significant policy gap and a lack of formal recognition by the state of the family’s role in care provision, especially given the visible reliance on families, particularly women, for unpaid care work.

Although family caregiving is not formally recognised in policy documents, the analysed sources showed that indirect support is visible through labour market-focused programs under the social sector. One document describing the Qurrah program stated that the initiative aimed to ‘support working women by subsidising childcare expenses for children at licensed centres’, thereby recognising the financial burden of caregiving. Similarly, a reviewed source describing the Wusool program indicated that it sought to ‘facilitate women’s access to workplaces through reduced transportation costs for private sector employees, particularly women and individuals with disabilities, by providing rides through authorised ride-hailing apps’. These programs, implemented under the Human Resources Development Fund (HADAF), were presented in the analysed webpages as initiatives that aimed to promote women’s workforce participation by easing caregiving-related logistical and financial challenges, thereby supporting job stability and acknowledging the caregiving responsibilities of women, especially working mothers and aim to reduce the burdens that restrict their participation in the labour market.

Notably, the reviewed documents showed that no codes or data were found linking the family directly to the healthcare responsibilities or positioning caregiving as part of a broader care infrastructure in the reviewed sources. The lack of formal acknowledgement in the analysed sources highlighted a significant policy gap in recognising and supporting unpaid caregiving roles, particularly those carried out by mothers, despite the visible reliance on families in sustaining care. Instead, the sources indicated that the state adopted an indirect approach by addressing caregiving through employment subsidies rather than care policies.

### Theme 5: the community’s role in filling care gaps was supportive yet inconsistent

The analysis of six community-sector sources revealed that the community played a complementary but uneven role in care provision. Half of the reviewed sources focused on healthcare, while the remaining focused on social and educational support. These sources consistently demonstrated through analysis how community-based organisations addressed unmet needs and acted as a fallback provider, offering care to underserved populations, including women, girls, and those requiring long-term health support, when state and market services fell short in terms of accessibility, scope, or cultural relevance.

In the healthcare sector, analysis has shown that Non-Governmental Organisations (NGOs) have collaborated with the National Home Health Care Foundation (NHHF) to deliver home-based medical services to patients with chronic or acute conditions who may require more frequent or personalised care than public services can offer. The analysis noted that this care was often delivered by multidisciplinary volunteer teams working in coordination with families, providing support tailored to local customs and community needs.

Community-led social care programs were highlighted in the reviewed sources as emphasising empowerment, especially of women and girls. One webpage stated that the initiative sought to ‘enable women to realise their potential and contribute to society’, illustrating how the community sector complements public efforts by filling service gaps, particularly in areas where institutional care is generalised or insufficiently personalised. Another analysed webpage described an initiative aimed at ‘providing a secure and nurturing environment where girls can explore their creativity through art’, which responded to developmental and educational needs not addressed in formal education institutions, while also empowering them with knowledge and economic opportunities. A community webpage highlighted a literacy program that ‘trained girls to teach their mothers to read and write’, demonstrating intergenerational disparities that may not be prioritised in state programs. While these programs contributed to national goals, such as improved living standards and social inclusion, the textual analysis revealed structural weaknesses, as several initiatives demonstrated that they relied on donations, volunteer labour, and informal networks, resulting in inconsistent service delivery and limited scalability. These discrepancies can be partially attributed to Saudi Arabia’s unique regulatory and historical background, where non-governmental organisations have historically operated through private charitable giving and donations, rather than as officially established service providers. This dependence on philanthropic funding and volunteer work creates susceptibility to resource variations and constrains their capacity for expansion or integration into national frameworks. According to Rogero-García’s classification system (45), this characteristic represents a distinctly Saudi adaptation of the semi-shared framework; community organisations address service gaps while maintaining loose ties to governmental structures, thereby strengthening state dominance while leaving inter-sectoral coordination poorly developed. Overall, the findings highlighted both the strength and fragility of the community sector, which adapted to unmet needs but lacked the necessary infrastructure and oversight to ensure equitable and sustained care.

## Discussion

The study examined the Saudi Arabian care economy by systematically exploring its structure, coordination, and challenges using Razavi’s care diamond framework, which analyses how care responsibilities are distributed among the state, market, community, and family sectors. Textual analysis revealed five interconnected themes reflecting systemic dynamics shaping care delivery (1): absence of the care economy (2); state as the primary architect of care (3); private sector participation in care (4); the family’s critical but overlooked role in care provision; and (5) the community’s role in filling care gaps is supportive yet inconsistent. The themes highlighted a fragmented, gendered, and inadequately integrated care system that is influenced by institutional arrangements, policy gaps, and sociocultural norms influencing Saudi Arabia’s evolving care landscape.

### Uneven representation of care diamond sectors

The study’s findings revealed that the four sectors of Razavi’s care diamond are represented unequally in Saudi policy discourse. The state played a dominant role in care provision across healthcare, education, and social care, both as a regulator and a service provider. This was evident in its responsibilities for developing national policies, allocating public funding, and overseeing the implementation of care-related programs. The textual analysis showed a clear imbalance in representation across the care diamond sectors. Of the 40 sources analysed, 27 were obtained from state institutions, reflecting the government’s strong visibility in care-related policy discourse and its role as a primary regulator and coordinator of care provision across sectors.

In contrast, the market sector was mentioned in seven sources and was primarily limited to independent healthcare providers, often operating without formal connections to public systems. The community sector was only slightly represented by six sources, reflecting voluntary, localised initiatives with no institutional support. The family sector was largely absent; no governmental documents formally identified families as care providers, despite their foundational role in caregiving. This dominance was further illustrated by budget allocations: 393 billion Riyals (10% of nominal GDP) directed to state ministries, compared to just 1.4 and 1.1% from the private sector, and 15 billion Riyals from the community (46). This imbalance indicates a care system where the state dominates both operationally and conceptually, while the roles of other sectors remain limited and largely unrecognised. As a result, the care diamond is misrepresented, with overconcentration of responsibility and authority within the state.

### Fragmentation and sectoral disconnection

Despite the state’s institutional dominance, the findings revealed a lack of coordination and integration across Razavi’s care diamond sectors, resulting in fragmented care service delivery. Private healthcare providers, such as Adeed and Fakeeh, offer home-based care operated independently of state services. Although public-private partnerships are intended to enhance service delivery, access remains stratified, with private providers focusing mainly on the rich and relying on nonprofit organisations and unpaid family caregivers to fill service gaps.

Likewise, community-based programs, such as those supporting literacy, home-based care, or girls’ education, ran parallel to government reforms rather than being integrated. This lack of structured interaction among the care diamond’s four sectors reinforces inefficiencies and duplication of services. For example, both state and market sectors provide similar eldercare services without integrated referral systems or planning, leaving beneficiaries to navigate through various providers to receive care, which can reduce access due to time, availability, and cost concerns (14). This is consistent with Andersen’s argument that fragmented welfare systems, where public and private roles are poorly defined or uncoordinated, result in gaps in access, reduced service quality, and widened inequalities (47).

### Unpaid care: a gendered and overlooked responsibility

The exclusion of family caregiving from national budgets, statistics, and policy frameworks highlights a significant gap within the family sector of Razavi’s care diamond, which is particularly notable given the gendered nature of unpaid care. In this framework, the family is recognised as a core provider of care, yet the analysis showed it is the least institutionally supported and formally acknowledged sector in Saudi Arabia’s care system. Although women are the primary caregivers in most households, this contribution remains unrecognised in policy documents, with no formal systems proposed to assist or redistribute the burden. As a result, unpaid family care primarily provided by women creates a visible gender burden. State-led initiatives, such as Qurrah childcare subsidy and Wusool transportation program, aim to support working mothers. However, these initiatives framed caregiving as a private, individualised responsibility rather than a shared or publicly supported responsibility. This framing excludes the family sector from coordinated policy planning and reinforces its isolation from the other three sectors, as initiatives focused on economic participation rather than being addressed through health or social care policies—thereby reinforcing the gendered distribution of care responsibilities (15). The analysis found no formal recognition of unpaid care across the reviewed documents, indicating a systemic gap in policy support for this essential contribution to family and social well-being. Women’s full economic involvement and household care remain unacknowledged in the absence of measures to recognise, reduce, or redistribute care work, such as flexible work arrangements, caregiver subsidies, or the inclusion of unpaid care in national accounts.

### Community care: essential but unsupported

The findings highlighted that community organisations in Saudi Arabia serve as fallback providers, addressing gaps left by the state and market. Their contributions, particularly in literacy programs for mothers, home-based care for patients with chronic conditions, and eldercare, are intended to alleviate the consequences of state dependency and reduce the burden on families. However, they often rely on voluntary labour and local funding, leading to inconsistent services. In Razavi’s care diamond, the community sector is expected to supplement and connect with the state, family, and market sectors to ensure integrated care provision. Nevertheless, the analysis revealed that community efforts functioned in parallel, rather than in collaboration, with formal systems, undermining the intersectoral linkages intended in the care diamond. This lack of integration hinders the community sector’s ability to enhance care delivery, particularly in underserved populations. This supports Putnam’s argument that over-reliance on social capital, such as informal networks without state backing, can create instability in care provision (48). Thus, while the community sector played an essential yet underrecognized role in bridging care gaps, its impact remained limited and uneven due to a lack of coordinated support from other sectors.

### Comparing with global care regimes: Rogero-García’s typology

To interpret the study’s findings within a broader global context, Rogero-García’s classification of care systems provides a useful comparative lens for further discussion (45). In his classification, care systems are classified into shared, semi-shared, and unshared based on the distribution of care responsibilities among the state, market, community, and family. In semi-shared systems, care is predominantly provided by families with minimal support of state services for the most dependent and market-based care is reserved for privileged groups like those seen in Latin America and Asia. Although Saudi Arabia’s care system structurally resembles the semi-shared model common in Asia. It presents significant differences in sectoral roles and responsibilities. In these systems, families, particularly women, are expected to provide most care while the contributions of the state and market remain minimal. Instead, the findings revealed a distinct deviation from this pattern. In Saudi Arabia, state support dominates, while the market, community, and family sectors play complementary but weakly integrated roles. This contrasts with the typical semi-shared model, in which families, particularly women, bear the primary burden of caregiving responsibilities with little state support. However, within the Saudi Arabian context, the state dominates care provision without incorporating market, family, and community sectors, creating a unique variant of the semi-shared model characterised by strong state capacity yet inadequate inter-sectoral coordination. This imbalance reflects strong state control with limited coordination among the care diamond sectors. As a result, the semi-shared classification may require contextual reinterpretation, especially in settings where state capacity is high but inter-sectoral coordination is largely absent.

### Vision 2030 and the missing care framework

Although the care economy is not explicitly referenced in Saudi Arabia’s Vision 2030 and related policy documents, its aspiration to develop a vibrant society, improve quality of life and increase women’s labour force participation align closely with the goals of inclusive and coordinated care provision. Vision 2030 emphasises interdependencies between ministries and the private sector to enhance access and quality of care. The current structure, however, lacks a guiding framework to connect and coordinate contributions from state, market, community, and family. The findings revealed fragmented roles and responsibilities: private providers and NGOs operate independently, despite their growing involvement in areas such as home-based healthcare, eldercare, and women’s empowerment; unpaid family care is excluded from national planning; and public initiatives often run in isolation.

Textual analysis revealed that the King Khalid Foundation’s report, a non-governmental source, explicitly mentions and discusses the care economy, underscoring its value for social and economic development. In contrast, state and market sources often framed care as either a health service or voluntary work, overlooking its broader economic and social contributions. As Rogero-García notes, the absence of unpaid care from policy and measurement frameworks distorts the true state of societal welfare (45). Similarly, Saudi Arabia’s exclusion of unpaid care from its national and GDP calculations mirrors global trends where women perform most of this invisible labour, mainly catering for children and the older population (48).

Even though it is not officially on the national agenda, the care economy, particularly unpaid care and grassroots initiatives, can support Vision 2030 if effectively integrated. Coordinating these efforts may enhance community resilience, close care gaps, and encourage workforce participation, particularly among women. Conversely, if left unaddressed, challenges such as market-driven exclusion, familial exhaustion, and inefficient service delivery will continue to challenge the realisation of Vision 2030’s inclusive development objectives.

Overall, the findings illustrate that Saudi Arabia’s care system is characterised by strong state dominance, weak integration among the market, community, and family sectors, and an imbalance clearly visible through Razavi’s care diamond. Despite substantial public investment and policy direction, the lack of coordination mechanisms resulted in duplication, fragmentation, and the marginalisation of unpaid and community-based care. While the system superficially resembles Rogero-García’s semi-shared model, Saudi Arabia’s huge state resources and limited intersectoral collaboration create a distinctive structure that requires contextual interpretation. Bridging these gaps through inclusive policy reform, formal recognition of unpaid care, and stronger cross-sectoral collaboration would be critical for achieving Vision 2030’s social and economic objectives.

### Study implication

The study’s findings suggest that Saudi Arabia needs to develop a formal policy framework for the care economy, ensuring coordinated participation across the state, market, community, and family sectors in line with Vision 2030 goals. Establishing a centralised, government-led body to supervise and coordinate care delivery at both national and local levels would be a critical step. Such a framework should include formal recognition of unpaid caregiving, particularly by families and community members, within national planning and budgeting processes. This approach could reduce care fragmentation, boost system efficiency, and improve the equity and quality of care across the Kingdom.

Although Saudi Arabia’s National Health Insurance is still in progress, lessons can be drawn from Qatar’s National Health Insurance Centre (NHIC) model, which ensures access to care while easing pressure on families (49, 50). Saudi Arabia’s care system remains state-led but lacks coordination, enabling care commodification and inconsistent community support, ultimately reducing access for low-income families and overburdening unpaid carers.

### Study limitation

This study relied solely on secondary data, primarily from government sources, with limited representation from the market and community sectors and none from the family sector. The high state presentation may amplify its role in the care economy, while fewer documents may underrepresent the perspectives of the grassroots challenges faced by market, community, and family participation. The absence of accessible data from the family, a key sector of Razavi’s care diamond, limited the study’s ability to capture the scope and impact of unpaid caregiving. Furthermore, some potentially relevant documents, particularly from non-state sectors, may not have been publicly accessible, further limiting the breadth of analysis. This gap reflects not the absence of care but the lack of formal documentation, highlighting the need for future research using primary data to capture these underrecognized care practices.

This study suggests that future research on the Saudi care economy should incorporate primary data to address gaps in insights from the family and community sectors. Specifically, ethnographic studies, along with methods such as semi-structured interviews and focus groups, are essential for capturing the lived experiences of unpaid caregivers and assessing their economic contributions. Although direct documentation from the family sector was lacking, references to family roles and challenges appeared in documents from the other sectors, offering partial insights that future studies can build upon.

## Conclusion

The care economy in Saudi Arabia can be characterised by structural invisibility, unequal access to services and sectoral fragmentation. Despite the state’s dominant role as the primary provider and regulator of care predominantly through healthcare, education, and social welfare, the care system lacks a unifying policy framework that conceptualises care as an integrated, cross-sectoral responsibility. This absence results in fragmented efforts across the state, market, community, and family sectors, compromising both efficiency and equity in care provision. Furthermore, the private sector’s involvement remains restricted and market-driven, often excluding vulnerable populations. In the meantime, families, women in particular, continue to shoulder a large portion of the caregiving burden without sufficient institutional assistance. The findings suggest a need for the Saudi government to formally recognise and include in policy documents the roles of the market, community, and family sectors, and to introduce the care economy as a policy priority to achieve Vision 2030’s goals of a vibrant society and thriving economy. Without such formal recognition and coordination of the care economy, efforts to enhance care provision will remain fragmented, potentially undermining the effectiveness of national development goals.

## Data Availability

The original contributions presented in the study are included in the article/supplementary material, further inquiries can be directed to the corresponding author.

## Footnotes

^1^In the Saudi context, “social care” refers to institutional and community-based services provided to vulnerable groups, including the older population, people with disabilities, orphans, and those requiring social welfare support (40).

## References

[1] General Authority for Statistics. (2024). General Authority for Statistics. Available online at: https://www.stats.gov.sa (Accessed April 10, 2025).

[2] World Health Organization. (2025). Global Health Observatory. Available online at: https://www.who.int/data/gho (Accessed May 6, 2025).

[3] Suleiman AK Ming LC. Transforming healthcare: Saudi Arabia’s vision 2030 healthcare model. J Pharm Policy Pract. (2025) 18:2449051. doi: 10.1080/20523211.2024.2449051, PMID: 39845746 PMC11753010

[4] HSTP. (2021). Health sector transformation program. Available online at: https://www.vision2030.gov.sa/en/explore/programs/health-sector-transformation-program (Accessed May 6, 2025).

[5] Alkhamis A Miraj SA. Access to health Care in Saudi Arabia: development in the context of Vision 2030. In: Laher I, editor. Handbook of healthcare in the Arab world. Cham: Springer (2021). 1629–60.

[6] Human Resources and Social Development. (2022). Achieving gender equality at work in Saudi Arabia. Available online at: https://www.hrsd.gov.sa/sites/default/files/2024-03/20220520_RBIC%20-%20KSA%20gender%20norms%20and%20BI%20report_3.pdf (Accessed September 29, 2025).

[7] Folbre N. Measuring care: gender, empowerment, and the care economy. J Hum Dev. (2006) 7:183–99. doi: 10.1080/14649880600768512

[8] International Labor Organization. (2024). Unpaid care work prevents 708 million women from participating in the labour market | International Labour Organization. Available online at: https://www.ilo.org/resource/news/unpaid-care-work-prevents-708-million-women-participating-labour-market (Accessed April 10, 2025).

[9] UN Women Arab States. (2020). English_PolicyBrief_Arab states. [policy brief]. Available online at: https://arabstates.unwomen.org/sites/default/files/Field%20Office%20Arab%20States/Attachments/Publications/2020/12/English_PolicyBrief_Arab%20States.pdf (Accessed September 29, 2025).

[10] Saudi Vision 2030 (2025). A thriving economy. Saudi Vis 2030 (2025). Available online at: https://www.vision2030.gov.sa/overview/pillars/a-thriving-economy (Accessed September 29, 2025).

[11] Qurrah (2019). Ministry of Human Resources and social development. Available online at: https://qurrah.sa (Accessed May 6, 2025).

[12] Human Resources Development Fund. (2017). Transportation support – Wusool. Available online at: https://www.hrdf.org.sa/en/products-and-services/programs/individuals/enable/wusool/ (Accessed April 10, 2025).

[13] Ministry of Human Resources and Social Development. (2025). Elderly care. Available online at: https://www.hrsd.gov.sa/en/care-about-you/care-elderly (Accessed October 1, 2025).

[14] King Khalid Foundation. (2023). KSA CARE ECONOMY choices for transformation and growth Prospect. Available online at: https://kkf.org.sa/en/ (Accessed November 3, 2024).

[15] DrA YA DrS HA. Fundamental shift in Saudi education system: increasing expenditure on private sector to improve learning. JEHS. (2024) 16:777–800. doi: 10.21608/jehs.2024.352153

[16] Folbre N. “Developing care: The care economy and economic development.” Women’s Economic Empowerment. Ottawa, ON: Routledge (2018).

[17] Razavi S. (2007). The political and social economy of Care in a Development Context Conceptual Issues, research questions and policy Options.

[18] Razavi S Staab S. Underpaid and overworked: a cross-national perspective on care workers. Int Labour Rev. (2010) 149:407–22. doi: 10.1111/j.1564-913X.2010.00095.x

[19] Vision 2030. (2023). Annual Report 2023. Available online at: https://www.vision2030.gov.sa/en/annual-reports (Accessed May 1, 2025).

[20] Ministry of Human Resources and Social Development. (2024). Women’s empowerment. Minist Hum Resour Soc Dev Available online at: https://www.hrsd.gov.sa/womens-empowerment (Accessed August 25, 2024).

[21] Perišić N Pantelić M. Care triangle or care diamond? The case of childcare and eldercare in Serbia. Rev Soc Polit. (2021) 27:323–45. doi: 10.3935/rsp.v28i3.1805

[22] Abe AK. (2010). The changing shape of the childcare diamond: the case of child and elderly care in Japan /: Aya K. Abe. Available online at: https://digitallibrary.un.org/record/684418 (Accessed November 3, 2024).

[23] Ochiai E. Care diamonds and welfare regimes in east and south-east Asian societies: bridging family and welfare sociology. Int J Jpn Sociol. (2009) 18:60–78. doi: 10.1111/j.1475-6781.2009.01117.x

[24] Duffy M Armenia A College R. (2021). A comparative analysis of 47 countries and territories.

[25] Razavi S. The return to social policy and the persistent neglect of unpaid care. Dev Chang. (2007) 38:377–400. doi: 10.1111/j.1467-7660.2007.00416.x

[26] Addati L Cattaneo U Esquivel V Valarino I. (2018). Care work and care jobs for the future of decent work.

[27] Peng I. (2019) The care economy: A new research framework. Available online at: https://sciencespo.hal.science/hal-03456901 (Accessed July 22, 2024).

[28] Yokobori Y Kiyohara H Mulati N Lwin KS Bao TQQ Aung MN. Roles of social protection to promote health service coverage among vulnerable people toward achieving universal health coverage: a literature review of international organizations. Int J Environ Res Public Health. (2023) 20:5754. doi: 10.3390/ijerph20095754, PMID: 37174271 PMC10177917

[29] Tisdell E.J Merriam S.B Stuckey-Peyrot H.L. (2025). Qualitative research: A guide to design and implementation. (5th ed.). Available online at: https://www.wiley-vch.de/en/areas-interest/humanities-social-sciences/education-12ed/higher-education-general-12ed2/assessment-evaluation-research-higher-education-12ed22/qualitative-research-978-1-394-26644-9 (Accessed May 6, 2025).

[30] Levitt HM Morrill Z Collins KM Rizo JL. The methodological integrity of critical qualitative research: principles to support design and research review. J Couns Psychol. (2021) 68:357–70. doi: 10.1037/cou0000523, PMID: 34043379

[31] Lim WM. What is qualitative research? An overview and guidelines. Australas Mark J. (2025) 33:199–229. doi: 10.1177/14413582241264619

[32] Brown N Collins J. Systematic visuo-textual analysis: a framework for analysing visual and textual data. Qual Rep. (2021) 26:1275–1290. doi: 10.46743/2160-3715/2021.4838

[33] Bellstam G Bhagat S Cookson JA. A text-based analysis of corporate innovation. Manag Sci. (2021) 67:4004–31. doi: 10.1287/mnsc.2020.3682, PMID: 19642375

[34] Braun V Clarke V. Using thematic analysis in psychology. Qual Res Psychol. (2006) 3:77–101. doi: 10.1191/1478088706qp063oa

[35] NVivo: Leading Qualitative Data Analysis Software. (2025). Lumivero. Available online at: https://lumivero.com/products/nvivo/ (Accessed July 6, 2025).

[36] Home Healthcare. (2025). Minist health Saudi Arab. Available online at: https://www.moh.gov.sa/en/Pages/Default.aspx (Accessed September 29, 2025).

[37] Saudi Health Council. (2009). Healthcare strategy in the kingdom. Available online at: https://shc.gov.sa/EN/Pages/default.aspx (Accessed April 10, 2025).

[38] Ministry of Human Resources and Social Development. (2025). Telework Program. Available online at: https://teleworks.sa/en/about-us/ (Accessed April 10, 2025).

[39] Ministry of Education. (2025). School health. Available online at: https://www.moe.gov.sa/en/education/generaleducation/Pages/SchoolHealth.aspx (Accessed May 6, 2025).

[40] Ministry of Human Resources and Social Development. (2025). Social services. Available online at: https://www.hrsd.gov.sa/en/care-about-you/social-protection (Accessed September 30, 2025).

[41] SABA (2025). SABA home care. Saba med Available online at: https://sabamedical.com/en/department/home-care/ (Accessed April 10, 2025).

[42] FAME. (2024). Child and mother care services. Available online at: https://famemed.com/service-4 (Accessed April 10, 2025).

[43] Enfield Royal Clinics. Private Care for the Elderly at home in Riyadh, Saudi Arabia. (2023) Available online at: https://www.enfieldroyalsaudia.com/private-care-for-the-elderly-at-home/ (Accessed April 10, 2025).

[44] National Platform. (2025). Private sector partnership. Available online at: https://my.gov.sa/en/content/partnership-private-sector#section-2 (Accessed October 1, 2025).

[45] Rogero-García J. (2012). Regions overburdened with care: continental differences in attention for dependent adults. Work Pap. Available online at: https://ideas.repec.org//p/fbb/wpaper/2012117.html (Accessed June 16, 2025).

[46] Ministry of Finance. (2023). State General Budget Statement. Available online at: https://mof.gov.sa/en/Pages/default.aspx (Accessed June 18, 2025).

[47] Esping-Andersen G. The three worlds of welfare capitalism. Reprint ed. Cambridge: Polity Press (1990). 248 p.

[48] Putnam R. (2000). Bowling Alone: The Collapse and Revival of American Community. 357 p.

[49] Vaca-Trigo I Scuro Somma L Stefanovié A. (2022). The care economy and unpaid work: Concepts and trends. Available online at: https://hdl.handle.net/11362/48230 (Accessed June 16, 2025.

[50] Al-Kaabi MH. Qatar’s National Health Insurance Company (NHIC): what happened, and what shall be done to develop the current social health insurance law. J Leg Ethical Regul Issues. (2021) 24:1.

