# Supplementary material for: Exploring Saudi Arabia’s care economy in health, education, and social care: a textual analysis using the care diamond framework
Source: Front Public Health. 2025 Oct 20;13:1688814. doi: 10.3389/fpubh.2025.1688814 (PMC12580255; doi:10.3389/fpubh.2025.1688814)
Supplement: Supplementary file 1 [file Table_1.docx]

**Appendix A: Sectors and Services provided**

**Table 1. Documents and Webpages Related to State**

| State entity | Type of services | Service description | Link to resource |
| --- | --- | --- | --- |
| Ministry of Health | Instant Medical Consultation Service | A service that allows all beneficiaries, and​ those in remote and rural areas to get instant medical consultations remotely | <https://www.moh.gov.sa/en/eServices/cards/Pages/Instant-Medical-Consultation.aspx> |
| Ministry of Health | Vaccination reminder for children | The Ministry sends reminders via phone or email one week before a child's vaccination, based on the new schedule. | <https://www.moh.gov.sa/en/eServices/cards/Pages/VaccinationsDescription.aspx> |
| Ministry of Health | Vaccination follow-up service | A service to track vaccinations, including flu, COVID, and Hajj vaccines, linked to the NVR system. | <https://www.moh.gov.sa/en/eServices/cards/Pages/Vaccination-Follow-up.aspx> |
| Ministry of Health | Modern Healthcare Model | Preventive care, safe childbirth, palliative care  Quality and efficiency, person-cantered care, integrated and interconnected service, available in care primary centres and homes | <https://www.moh.gov.sa/en/Ministry/About/Pages/Definition-Modern-Healthcare-Model.aspx> |
| Ministry of Health | Support Groups initiative | It connects patients with chronic or rare diseases and their families to healthcare practitioners for physical, psychological, and social support. It facilitates the formation of groups where members share experiences and offer mutual support under the guidance of a health educator. | <https://www.moh.gov.sa/en/Ministry/Projects/Support-Groups/Pages/default.aspx> |
| Ministry of Education | Women Empowerment |  | <https://moe.gov.sa/en/aboutus/nationaltransformation/pages/womenandtheministry.aspx> |
| Ministry of Education | Early Childhood |  | <https://www.moe.gov.sa/en/education/generaleducation/Pages/Kindergarten.aspx> |
| Ministry of Education | ​​​​People with Disabilities |  | <https://www.moe.gov.sa/en/education/highereducation/Pages/PeopleWithSpecialNeeds.aspx> |
| Ministry of Education | School health |  | <https://www.moe.gov.sa/en/education/generaleducation/Pages/SchoolHealth.aspx> |
| Ministry of Education | Regulations for International and Private Schools |  | <https://www.moe.gov.sa/en/education/generaleducation/Pages/Reg-priv-int-schools.aspx> |
| Ministry of Education | Partnership with the private sector |  | <https://moe.gov.sa/en/aboutus/aboutministry/DecisionsAndPartnerships/Pages/PartnershipPrivateSector.aspx> |
| Ministry of Education | Partnerships with the Civil Society Organizations: |  | <https://www.moe.gov.sa/en/aboutus/aboutministry/decisionsandpartnerships/pages/localpartnerships.aspx> |
| Ministry of Education | Completed projects |  | <https://www.moe.gov.sa/en/knowledgecenter/projectsinitiatives/Pages/completedprojects.aspx> |
| Ministry of Human Resources and Social Development | Home Health Care for the Elderly | A team of professionals, including a doctor, psychologist, and caregivers, provides health, psychological, and physical care for the older population within the family | <https://www.my.gov.sa/wps/portal/snp/servicesDirectory/servicedetails/s9137> |
| Ministry of Human Resources and Social Development | Women's leave  (delivery, maternity, bereavement) | These services are granted for all working women in both government and private sectors however the  maternity and delivery leaves duration may differ but  the Bereavement Leave is the same as per Islamic rules  for Muslim women | <https://www.hrsd.gov.sa/en/knowledge-centre/articles/64410> |
| Human Resources Development Fund (HADAF)- Ministry of Human Resources and Social Development | Qurrah Subsidy Program | National initiative of the Child Welfare Authority, the Human resources Development Corporation to support and empower Saudi working women to join the labour market and invest in it and reassuring of the care provided to her children with her support in the care provided. | <https://qurrah.sa/aboutsubsidy> |
| Human Resources Development Fund (HADAF)- Ministry of Human Resources and Social Development | Women's Transportation Support Program "Wusool" | -Facilitating transportation of female employees in the private sector  -Reducing transport costs  -Stability in the labour market | <https://www.hrdf.org.sa/en/programs/individuals/enable/wusool/> |
| Ministry of Human Resources and Social Development | Telework program | -Teleworking (virtual workplace)  -Flexible work environment  -Foster new cultures  -New job opportunities  -Overcome physical obstacles  -Women can take care of children during work hours | <https://teleworks.sa/en/about-us/> |
| Family Affairs Council- Ministry of Human Resources and Social Development | -Childhood committee  -Women’s committee  -Elderly committee |  | <https://fac.gov.sa/en/about-fac/#fac_about_committees> |
| Ministry of Human Resource and Social Development | -Social protection  -Health and safety in the workplace  -Child support  -Skills and training | -Youth and women empowerment  -Empowering people with disabilities  -Safe workplace  -Social protection  -Juvenile care  -Elderly care  -Resilient and thriving society  -Addressing citizen needs | <https://www.hrsd.gov.sa/en/social-protection> |
| Ministry of Human Resources and Social Development | Manual of Women's Employment in  the Private Sector | -Regulation manual for women's work in shops and family entertainment venues  -Security of women in shops  -Comfort and safety ta work | <https://eduschool40.blog/wp-content/uploads/2021/10/%D8%AF%D9%84%D9%8A%D9%84-%D8%B9%D9%85%D9%84-%D8%A7%D9%84%D9%85%D8%B1%D8%A7%D8%A9-%D9%81%D9%8A-%D8%A7%D9%84%D9%82%D8%B7%D8%A7%D8%B9-%D8%A7%D9%84%D8%AE%D8%A7%D8%B5.pdf> |
| Ministry of Human Resource and Social Development | Women’s Empowerment |  | <https://www.hrsd.gov.sa/en/womens-empowerment> |
| Family Affairs Council- Ministry of Human Resource and Social Development | Life Skills Guide  (Public Health chapter) | -Life skills for physical fitness  -Promotes wellbeing and quality of life  -Raises body immunity  -Stress management | <https://fac.gov.sa/en/guidelines-posts/%d8%af%d9%84%d9%8a%d9%84-%d8%a7%d9%84%d8%ad%d9%8a%d8%a7%d8%a9-%d9%85%d9%87%d8%a7%d8%b1%d8%a9-2024/> |
| Vision 2030 report | Through multiple vision realization programs |  | <https://www.vision2030.gov.sa/en/annual-reports> |
| Healthcare Sector Transformation Program (HSTP) | Vision Realization Programs  Under vision 2030 | Achieving government excellence, high employment rate for disabled people, more women joining the workforce | <https://www.vision2030.gov.sa/media/0wop2tds/hstp_eng.pdf> |
| King Khalid Foundation | KSA Care Economy Report on transformation and growth prospects | -Complex care system  -Workforce readiness  -Gaps to access to quality services  -Funding of healthcare sector  -Policy supports for population increase an ageing people  -Transformation through integration and coordination of care  -Reduced costs, improved quality, accessible services, accommodate rising population, transformed healthcare for Vision 2030 | <https://www.kkf.org.sa/media/khkln1nt/care_report_05_en-1.pdf> |
| Saudi Health Council | Healthcare Strategy in the Kingdom | -Empowerment -Transparency  -Responsibility  -Stimulus  -Knowledge exchange  -National centres available  -Health awareness | <https://shc.gov.sa/EN/Pages/default.aspx>  <https://shc.gov.sa/Arabic/New_Strategy/Documents/Strategy%20Book.pdf> |

**Table 2. Webpages Related to Market**

| Market entity | Type of services | Service description | Link to resource |
| --- | --- | --- | --- |
| Fakeeh | Home healthcare | 24-hour support services, palliative healthcare, medical assistance Link  to chronic patients, comprehensive medical care | <https://en.fakeeh.care/ambulatory-care-services/home-health-care>  <https://en.fhhc.fakeeh.care/> |
| ADEED | Home healthcare | Home medical care, laboratory tests, nursing services, care Link assistant, doctor visit at home, lab test at home, dietician services, physiotherapy at home | <https://adeed.com/en/home> |
| SABA | Home healthcare | Newborn care, home nursing and elderly care, medical examination services, laboratory services, radiography services, dental services. | <https://sabamedical.com/en/department/homecare/> |
| FAME | Home healthcare | Birth assistance services, pregnancy tests, maternal health monitoring, caesarean care services, accompaniment for mother and  child after birth, breastfeeding training for mothers. | <https://famemed.com/service-4> |
| Enfield Royal Clinic | Home healthcare for the elderly | Private care for the elderly at home, caregiving to the elderly, companionship and social support | <https://www.enfieldroyalsaudia.com/private-care-for-the-elderly-at-home/> |
| Ministry of Health | Health Sector Partnerships |  | <https://my.gov.sa/en/content/partnership-private-sector#section-3> |
| National Platform GOV.SA | **Education Sector Companies** |  | <https://my.gov.sa/en/content/partnership-private-sector#section-2> |

**Table 3. Webpages Related to Community**

| Community entity | Type of services | Service description | Link to resource |
| --- | --- | --- | --- |
| Girls Hali | Girls Hali NGO | Social development, empowering women and girls | <https://fatahhali.sa/> |
| ALNAHDA | Social and economic empowerment | Economic and social empowerment of women | <https://www.alnahda.org/about> |
| Civil Associations NGOs | Community services | Family development, housing services. enhancing the role of the family in developing, improving standard of living | <https://fac.gov.sa/web/main_dept/22> |
| We Care | Social services | Social work in medical field, school, public assistance, social insurance | <https://www.nhhcf.org/en/services_details.php?service_id=9> |
| Gulf Girl (Health Committee) | Charity for women | Raising health of families in  cooperation with government and civil authorities | <https://fatatalkhaleej.org.sa/committees/healthCommittee> |
| Ministry of Health | Community Partnership Agreement with Magrabi Hospitals and Centers |  | [Community Partnership Agreement with Magrabi Hospitals and Centers](https://www.moh.gov.sa/en/Ministry/MediaCenter/News/Pages/News-2020-11-19-007.aspx) |
